# New-onset stroke on the risk of hip fracture: the Kailuan cohort study in China

**DOI:** 10.1186/s12889-023-15787-5

**Published:** 2023-05-22

**Authors:** Nan Zhang, Lu Guo, Yaohui Yu, Shuohua Chen, Lishu Gao, Xiaoli Hou, Faming Tian, Shouling Wu

**Affiliations:** 1 Department of orthopedics, Kailuan General Hospital, Tangshan, Hebei China; 2 the School of Public Health, North China University of Science and Technology, Tangshan, Hebei China; 3 Department of Cardiology, Kailuan General Hospital, Tangshan, Hebei China; 4 Department of Endocrinology, Tangshan People’s Hospital, Tangshan, Hebei China

**Keywords:** Stroke, Hip fracture, Risk, Cohort study

## Abstract

**Purpose:**

Stroke is a documented risk factor for hip fracture(HF). However, no data is currently available on this issue in mainland China, we therefore assessed the risk of hip fracture after new-onset stroke using a cohort study.

**Methods:**

This study included 165,670 participants without a history of stroke at baseline from the Kailuan study. All participants were followed biennially until December 31, 2021. During follow-up, a total of 8,496 new-onset stroke cases were identified. For each case subject, four control subjects was randomly selected, matched for age (± 1 years) and sex. The final analysis comprised 42,455 pair-matched cases and controls. A multivariate Cox proportional hazard regression model was used to estimate the effect of new-onset stroke on the risk of hip fracture.

**Results:**

During an average follow-up of 8.87 (3.94) years, a total of 231 hip fracture cases occurred, 78 cases in the stroke group and 153 cases in the control group, with incidence rates of 1.12 and 0.50 per 1000 person-years, respectively. The cumulative incidence of the stroke group was higher than that of the controls (*P* < 0.01). The adjusted hazard ratio (95% confidence interval) of hip fractures in the stroke group was 2.35 (1.77 to 3.12) (*P* < 0.001) to controls. After stratifying by gender, age, and body mass index, the higher risk was revealed in female (HR 3.10, 95 CI: 2.18 to 6.14, *P* < 0.001), age < 60 years old (HR 4.12, 95% CI: 2.18 to 7.78, *P* < 0.001), and non-obesity (BMI<28 kg/m^2^) (HR 1.74, 95% CI:1.31 to 2.31, *P* < 0.001) subgroup.

**Conclusions:**

Stroke significantly increases the risk of hip fracture, strategy for protecting stroke patients from falls and hip fractures should be emphasized in poststroke long-term management, particularly the female, age < 60 years old, and non-obese patients.

**Supplementary Information:**

The online version contains supplementary material available at 10.1186/s12889-023-15787-5.

## Introduction

Stroke is a common cerebrovascular disease and the second most common cause of death and long-term disability worldwide [1]. The latest American Heart Association update on stroke statistics reported 89.13 million cases of stroke worldwide from 1990 to 2020 [2]. The latest research [3] shows that an estimated 3.94 million new strokes occur in China every year. The prevalence of stroke is 442.1 per 100,000, and there has been an increase in the incidence of stroke in recent years in China [4]. The sequelae of stroke include impaired balance, cognitive impairment, aphasia, and hemiplegia [5]. Stroke reduces quality of life for patients and imposes an economic burden on society and families. Stroke also increases the risk of falls [6] and is an important risk factor for hip fracture (HF).

A previous study [7] revealed that the risk of HF after stroke is 1.5-fold to 4-fold than that in the general population. In a prospective cohort from Sweden [8], the risk of HF was higher in stroke patients than in healthy individuals (hazard ratio 2.29). Lin et al. [9] also found that after adjustment for sex, age, and medications used, HF was more likely to occur in stroke patients than in controls matched for age and sex (hazard ratio 1.89). However, previous studies have mainly focused on estimating the risk of HF in individuals with prevalent stroke, rather than those with new-onset stroke, and did not account for the duration of stroke, which may affect the risk of HF.

Although the number of stroke cases is high in China [10], there is a paucity of data on the relationship between stroke and HF. In this study, we investigated the effect of new-onset stroke on the risk of HF in the Chinese population based on data from the Kailuan Cohort Study [11].

## Methods

### Data source and study setting

The rationale for the Kailuan Cohort Study and its design and methodology have been described in detail elsewhere [12]. In brief, 171,086 adults aged 18–98 years were enrolled from 11 hospitals affiliated with the Kailuan Group between June 2006 and December 2020 and participated in a biennial questionnaire interview and clinical and laboratory examinations. The Kailuan cohort was established in the Kailuan community in Tangshan, Hebei Province, China. The study was registered as ChiCTR-TNC-110,011,489 at http://www.chictr.org.cn/index.aspx with the registration number 110,011,489 [11]. The present research was approved by the Ethics Committee of Kailuan General Hospital and conducted in accordance with the Declaration of Helsinki.

### Data collection

Data were obtained for new-onset stroke cases from the most recent physical examination before the stroke event. Additional information on history of stroke was collected via the biennial questionnaire in the Kailuan Cohort Study since 2006. The provincial vital statistics offices collect information on deaths and the Kailuan database contains demographic information, personal medical history, and lifestyle data, which were collected via a face-to-face questionnaire interview with each study participant. These data were updated at 2-year intervals thereafter. Anthropometric and clinical data, including height, weight, heart rate, and blood pressure, were measured by trained staff using standardized procedures that have been reported in detail previously [13]. All study participants fasted for 8–12 h overnight before the physical examination, at which time 5 mL of venous blood was drawn from the vein in the antecubital fossa. The blood samples were analyzed using an automated biochemical analyzer (7600; Hitachi, Tokyo, Japan). All laboratory tests performed have already been reported [11].

### Study population

Between 2006 and December 2020, 166,110 enrolled individuals (excluding those with a history of stroke at their first physical examination) participated in a biennial physical examination, including laboratory and clinical examinations and a questionnaire interview. Additionally, all participants were follow-up annually to check for cardiovascular and cerebrovascular events, including stroke.

**Fig. 1:**
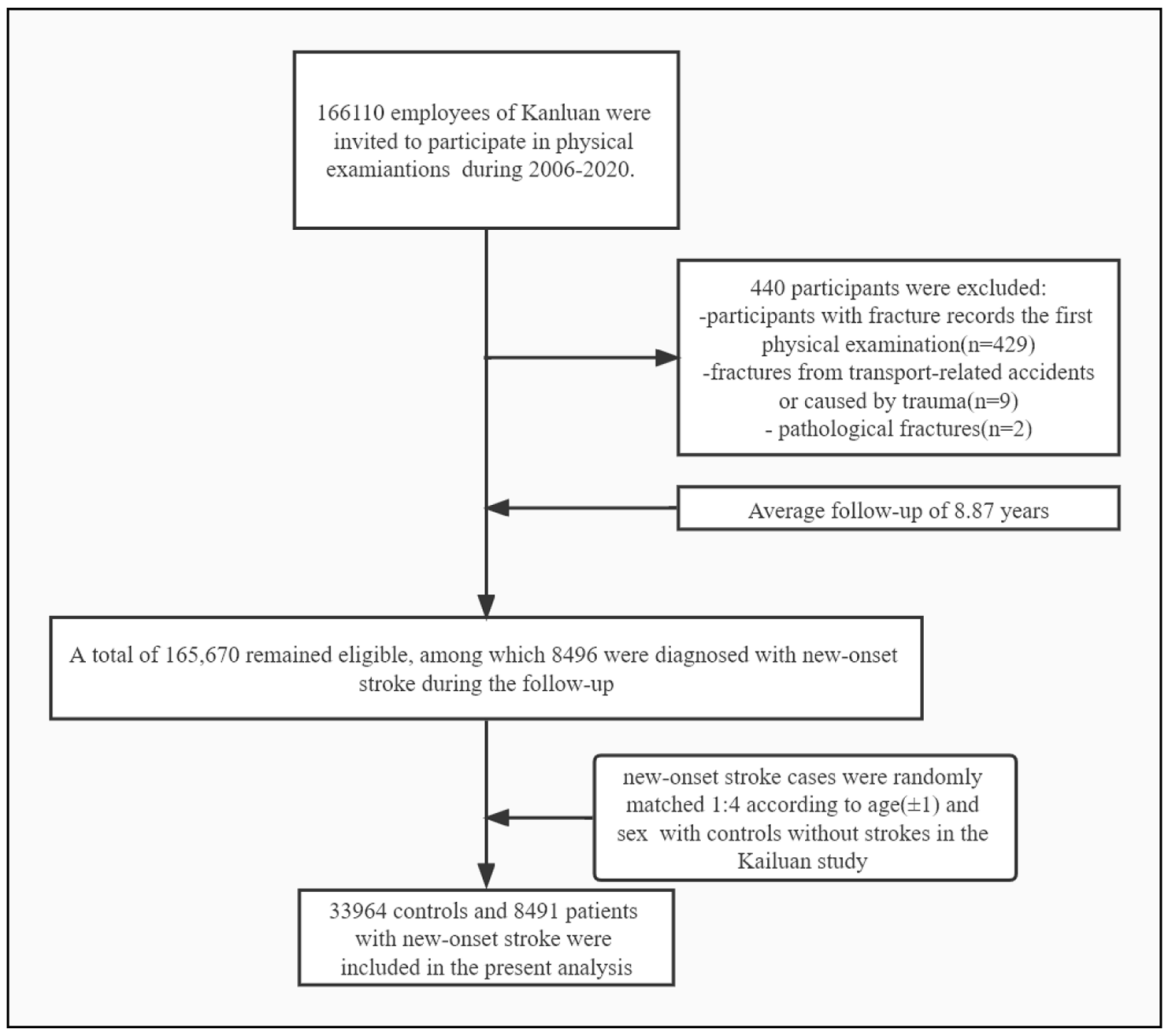
Selection scheme for the study population

To investigate the effect of stroke on the risk of HF, Stroke cases that met the following criteria were included: participation in annual health check-ups and surveys between 2006 and 2018; diagnosis of new-onset stroke; and willing to provide written informed consent. Matched controls participants based on the following criteria: physical examination performed on the same index date as that for the case; same sex and similar age (± 1 year); no diagnosis of stroke at the time of enrollment or during follow-up; and willing to provide written informed consent. For the current study, after exclusion of 429 participants with a history of fracture recorded at the first physical examination, nine participants with a fracture sustained in a transport-related accident or as a result of trauma, and two participants with pathological fractures, leaving 165,670 participants who met the inclusion criteria. Of the 165,670 participants without stroke at baseline, 8,496 participants were diagnosed with new-onset stroke (International Classification of Diseases ninth and tenth edition code I63 or I64) during follow-up. Ultimately, data for 42,455 pair-matched cases and controls were analyzed (Fig. 1).

### Definitions

#### New-onset stroke

New-onset stroke was defined as an ischemic or hemorrhagic stroke that occurred during follow-up. A hemorrhagic stroke included intracerebral hemorrhage and subarachnoid hemorrhage. In our study, we followed a standardized protocol to identify potential fatal and non-fatal cerebrovascular disease cases using the International Classification of Diseases (ICD)-10th Revision [14], as previously described [15, 16]. The outcome was further verified by cross-checking discharge summaries from the 11 participating hospitals, medical records from medical insurance, and self-report questionnaires from biennial follow-up surveys. For potential stroke cases identified by the ICD code and/or questionnaire, a panel of three physicians reviewed the medical records. Non-fatal strokes were defined as the sudden onset of focal neurological deficit with vascular mechanism lasting more than 24 h. Fatal strokes were confirmed by medical records, autopsy reports, or death certificates citing stroke as the cause. Stroke diagnosis was made using the World Health Organization criteria, combined with brain computed tomography or magnetic resonance imaging for confirmation [17]. The stroke index date was defined as the date of the first hospitalization for treatment of stroke.

#### Hip fracture

HF was diagnosed if any of the following ICD-10 codes were found in the national hospital discharge register [18]: S72.2 (subtrochanteric fracture), S72.1 (pertrochanteric fracture), and S72.0 (fracture of neck of femur). Subtrochanteric fractures were defined as fractures occurring within 5 cm distal to the lesser trochanter [19, 20]. Pertrochanteric fracture of femur refers to the fracture from the base of femoral neck to the level above the lesser trochanter [21]. Fracture of neck of femur refer to the fractures occurring between the femoral head and the basal part of the femoral neck [22]. The Municipal Social Insurance Institution database was used to obtain HF data. To ensure the accuracy of diagnosis of HF, basic patient information, imaging data (radiographs, computed tomography scans, or magnetic resonance images), and details of accidents resulting in HF contained in the medical records were reviewed.

### Study outcomes and follow-up

Taking the diagnosis time of new-onset stroke as the starting point, each participant’s observation time ends at the time of the incident HF event, of death, and ends (December 31, 2021) to follow-up, two or more hip fracture events are counted as one hip fracture event, whichever based on the time of the first event. The occurrence of two or more HFs was counted as one case of HF. Briefly, information on HFs was obtained from the Municipal Social Insurance Institution and discharge registers of all 11 affiliated hospitals in the Kailuan group annually from 2006 onwards.

For each new-onset stroke case, four controls matched for sex and age (± 1 year), were randomly selected from adults who underwent a physical examination in the same year and did not experience a stroke during follow-up. The follow-up duration was conducted from the date of diagnosis of stroke and the same year for the matched controls [13]. For example, when a stroke occurred in a 60-year-old woman in 2008, four female controls who were aged 59–61 years and underwent a physical examination in 2008 and did not have a stroke during follow-up were randomly selected from the study population. and those five participants had been monitored since 2008. Successfully matched controls will not be included in subsequent matches.

### Covariates

Body mass index (BMI) was calculated by dividing body weight in kilograms by height in meters squared. Obesity was defined by a BMI of ≥ 28. Alcohol consumption was defined as having ingested 100 mL (alcohol content ≥ 50%) per day for more than one year and currently not abstaining from alcohol. Current smoking was defined as having smoked at least one cigarette per day for at least one year. Regular physical activity was defined as at least half an hour of moderate physical activity five or more times per week or at least 20 min of vigorous physical activity at least three times per week, including during the past week. Per capita monthly household income was considered low if less than 1,000 yuan (RMB). Dietary salt intake was considered high if ≥ 10 g/day. Hypertension was defined as a systolic blood pressure ≥ 140 mmHg, diastolic blood pressure ≥ 90 mmHg, current use of antihypertensive medication, and/or a history of hypertension [13]. Use of antihypertensive, lipid-lowering, or hypoglycemic medication was defined as starting any of these agents at the time of diagnosis of stroke or any point during the entire period of follow-up.

### Statistical analysis

Data that were distributed normally are shown as the mean ± standard deviation if continuous and as the number and percentage if categorical. Baseline characteristics were compared between the two groups using the Student’s *t*-test for continuous variables and the chi-squared test for categorical variables. Non-normally distributed data were compared between the groups using the Wilcoxon rank sum test and are shown as the median (interquartile range). The cumulative incidence of HF was calculated using the Kaplan-Meier method and compared using the log*-*rank test. Schoenfeld residuals were used to test the proportional hazards hypothesis before constructing a Cox proportional hazards regression model to calculate hazard ratios and 95% confidence intervals (CIs) for incident HF in stroke cases vs. controls. The first multivariate model was adjusted for current smoking (yes/no), current alcohol consumption (yes/no), dietary salt intake (high/low), regular physical activity (yes/no), nature of work (manual labor/sedentary), income (high*/*low), and BMI (≥ 28/<28). The second multivariate model was further adjusted for triglyceride, low-density lipoprotein cholesterol, and C*-*reactive protein levels and use of antihypertensive (yes/no), lipid-lowering (yes/no), and hypoglycemic (yes/no) medication. The third multivariate model was adjusted for history of myocardial infarction (yes/no) and history of atrial fibrillation (yes/no). We used multiple imputation by chained equations to impute missing value for covariates [23]. To determine whether there were differences in the risk of endpoint events according to age, sex, and BMI, a multiplicative interaction term with these three variables was constructed in the Cox model for stroke cases and controls to explore the potential interaction using the likelihood ratio test. Interactions with a *p*-value < 0.05 were considered statistical differences existed. The Cox model was repeated after stratification by age, sex, and BMI. The results of the subgroup analyses are presented as forest plots with HRs and 95% CIs.

Sensitivity analyses were then conducted to assess the robustness of the study findings. First, in the full cohort (before matching), we respectively excluded participants with missing covariates data, study participants using antihypertensive, hypoglycemic, or lipid-lowering medication followed by those with a history of malignancy to minimize the influence of these variables on the results. Second, to reduce confounding factors and enable direct comparison between stroke patients and controls, we employed propensity score matching in our sensitivity analysis. We matched strokes patients with controls using propensity score (1:4) based on various factors such as sex, age, current smoking, current drinking, high salt diet, physical activity, job nature, income, BMI, triglycerides, low-density lipoprotein cholesterol, C-reactive protein, use of antihypertensive drugs, lipid-lowering drugs, hypoglycemic drugs, history of myocardial infarction, history of atrial fibrillation, hypertension, and diabetes. Finally, the analyses were repeated after including a Fine-Gray competing risks hazard regression analysis with death as a competing event in the Cox proportional hazards model. All statistical analyses were performed using SAS version 9.4 software (SAS Institute Inc, Cary, NC). A *p*-value < 0.05 was considered statistical differences existed.

## Results

### Baseline characteristics

The baseline characteristics of the study participants are presented in Table 1. The mean age of the 33,964 controls and 8,491 cases was 62.57 ± 10.11 years and 62.58 ± 10.11 years, respectively; 89.61% of the study participants were male. Compared with controls, the new-onset stroke cases had higher total cholesterol, triglyceride, low-density lipoprotein cholesterol, and fasting blood glucose levels, higher C-reactive protein, and a lower high-density lipoprotein cholesterol level. The new-onset stroke group also had higher proportions of current smoking, high dietary salt intake, obesity, manual labor, history of atrial fibrillation, history of myocardial infarction, diabetes, hypertension, and use of antihypertensive, lipid-lowering, and hypoglycemic medications, and lower rates of current alcohol consumption, regular physical activity, and education to at least high school level (all *p* < 0.05). There was no statistical differences in the proportion with a high income or the proportion with a history of malignancy between controls and new-onset stroke cases (*p* > 0.05).

**Table 1:** Baseline characteristics of participants

Variables	Controls	Stroke patients	*P* value
Participants, n	33,964	8491	—
Age, years	62.57 ± 10.11	62.58 ± 10.11	1.000
Male,n(%)	30,436(89.61)	7609(89.61)	1.000
Current smoking, n(%)	12,702(37.40)	3482(41.01)	<0.001
Current drinking, n(%)	11,035(32.49)	2624(30.90)	0.005
High salty diet, n(%)	3012(8.87)	894(10.53)	0.048
BMI(≥ 28 kg/m^2^), n(%)	5430(15.99)	1794(21.03)	<0.001
Physical activity, n(%)	5572(16.41)	1318(15.52)	0.092
Manual labor, n(%)	30,310(89.24)	7818(92.07)	<0.001
High-income level, n(%)	3539(10.42)	832(9.80)	<0.001
≥Senior high school, n(%)	5194(15.29)	1032(12.15)	<0.001
TC mmol/L	4.99 ± 1.16	5.08 ± 1.32	<0.001
TG mmol/L M(P_25_,P_75_)	1.26(0.89,1.89)	1.38(0.98,2.12)	<0.001
HDL_C mmol/L	1.48 ± 0.44	1.45 ± 0.44	<0.001
LDL_C mmol/L	2.66 ± 0.90	2.73 ± 0.93	<0.001
FBG mmol/L	5.80 ± 1.71	6.38 ± 2.49	<0.001
hs-CRP,mg/L(P25,P75)	1.20(0.50,2.97)	1.40(0.60,3.43)	<0.001
Hypertension, n(%)	18,712(55.09)	6532(76.93)	<0.001
Diabetes, n(%)	4958(14.60)	2084(24.54)	<0.001
History of myocardial infarction, n(%)	700(2.06)	225(2.65)	<0.001
History of atrial fibrillation, n(%)	740(2.18)	317(3.73)	<0.001
History of malignant tumor, n(%)	405(1.19)	94(1.11)	0.514
Antidiabetic treatment, n(%)	2194(6.46)	894(10.53)	<0.001
Lipid-lowering treatment, n(%)	3063(9.02)	1779(20.95)	<0.001
Antihypertensive treatment, n(%)	10,958(32.26)	4515(53.17)	<0.001

*Note: Data are presented as mean ± standardized deviation, median (p_25_, p_75_), or n(percentage). BMI, body mass index; FBG, fasting blood glucose; LDL-C, low-density lipoprotein cholesterol; HDL-C, high-density lipoprotein cholesterol; hs-CRP, high-sensitivity C reactive protein; TG, triglyceride; TC, total cholesterol

### Incidence of hip fracture in controls and stroke cases

**Fig. 2:**
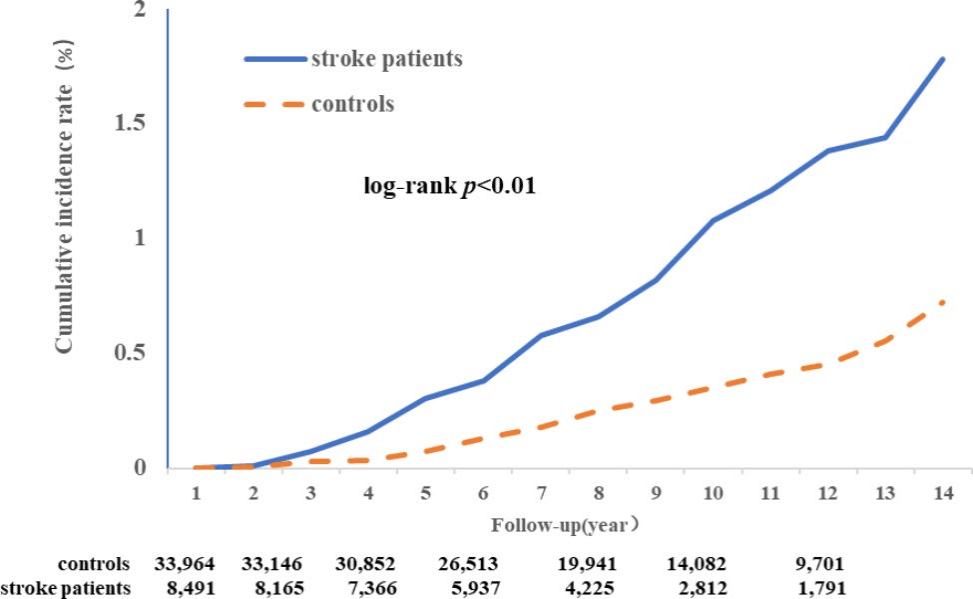
The Kaplan-Meier curves for the Cumulative Incidence of hip fracture in Stroke patients Compared with the Matched controls

During a median follow-up of 8.87 ± 3.94 years, we documented 231 HF events, of which 78 were in stroke cases and 153 were in controls. The incidence of HF was 1.12 per 1000 person-years in stroke cases and 0.50 per 1000 person-years in controls. The total follow-up time for the controls and strokes group was 327,200 person-years and 69,573 person-years(Table 2). The Kaplan-Meier estimate for the 12-year observed cumulative incidence of HF was 1.44% for new-onset stroke cases and 0.64%, for controls (*p* < 0.01, log*-*rank test; Fig. 2).

**Table 2:** .Hazard Ratios and 95% Confidence Intervals for the Incidence of hip fracture for Stroke patients Compared With the Matched controls

	Events/Participants (N)	Follow-up duration (PYs)	Incidence rate (per1000 PYs)		Model 1			Model 2				Model 3
					HR(95%CI)*p* value		HR(95%CI)*p* value				HR(95%CI)*p* value	
Controls	153/33,964	307,200	0.50		1.00		1.00			1.00		
Stroke patients	78/8491	69,573	1.12	2.36(1.80–3.02) < 0.001		2.34(1.76–3.10) < 0.001			2.35(1.77–3.12) < 0.001			

*Note: HR and 95%CI of hip fractures were calculated in the stroke patients compared to controls; incidence density rate = number of incident cases/person-years × 1000; PY, person-years; HR, hazard ratio. Model 1: adjusted for current smoking, current drinking, high salt diet, physical activity, job nature, income, and BMI. Model 2: adjusted for model 1 plus triglycerides, low-density lipoprotein cholesterol, C*-*reactive protein, use of antihypertensive drugs, lipid-lowering drugs, and hypoglycemic drugs. Model 3: adjusted for model 2 plus history of myocardial infarction, and history of atrial fibrillation

A multivariate analysis was performed after adjustment for current smoking status, current alcohol consumption status, dietary salt intake, amount of physical activity, type of work, income, BMI, C*-*reactive protein, triglyceride, and low-density lipoprotein cholesterol levels, use of antihypertensive, lipid-lowering, or hypoglycemic medication, history of myocardial infarction, and history of atrial fibrillation. The risk of HF remained higher in stroke cases than in controls (adjusted HR 2.35, 95% CI 1.77–3.12; Table 2).

### Subgroup analysis of hip fracture risk in stroke cases

**Fig. 3:**
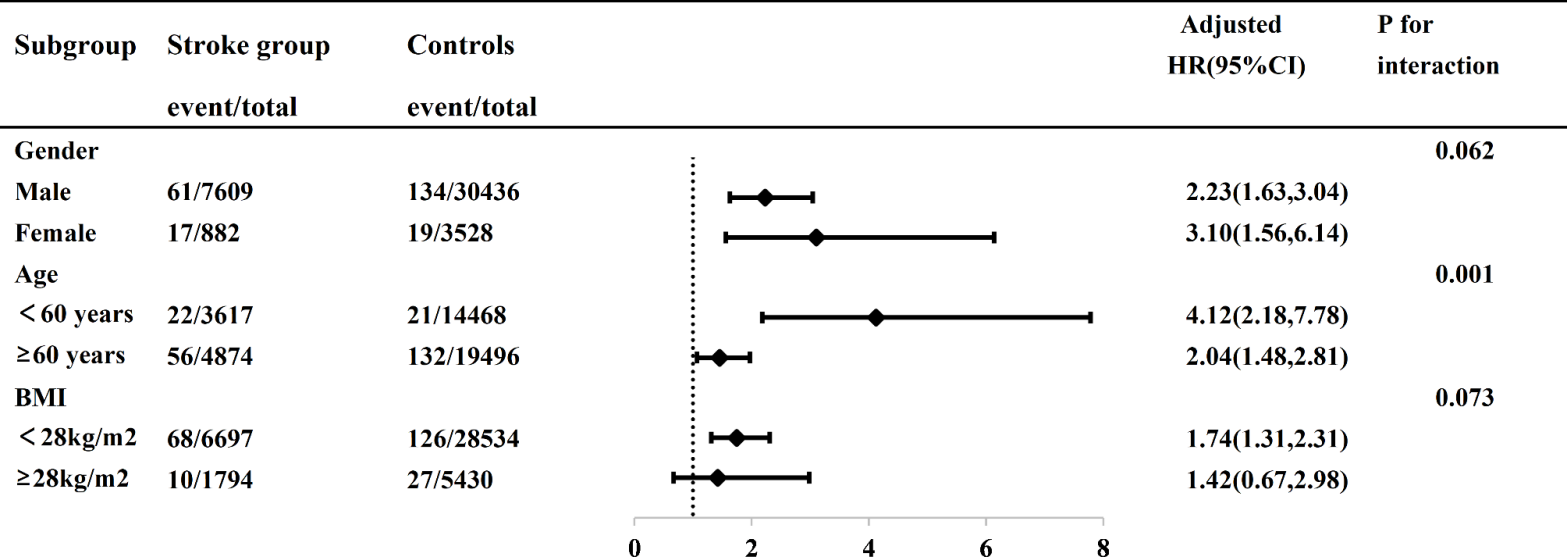
**Subgroup analysis for the Incidence of HF in Stroke patients Compared with the Matched controls** *Note; HR, hazard ratio; aHR indicates adjusted hazard ratio and CI, confidence interval. Adjusted for current smoking, current drinking, high salt diet, physical activity, job nature, income, BMI, triglycerides, low-density lipoprotein cholesterol, C*-*reactive protein, use of antihypertensive drugs, lipid-lowering drugs, hypoglycemic drugs, history of myocardial infarction, and history of atrial fibrillation

Potential interactions of stroke with sex, age, and BMI were explored in a Cox proportional hazards model, and the interaction effects were found to be 0.062, 0.001, and 0.073, respectively (Fig. 3). Stratified analyses were performed for age (*<* 60 years vs. *≥* 60 years), sex (female vs. male), and BMI (obese vs. non-obese). After adjustment for confounders, stroke cases had a higher risk of HF regardless of sex (female: HR 3.10, 95% CI 1.77–3.12; male: HR 2.23, 95% CI 1.77–3.12). Younger stroke cases also had a higher risk of HF (< 60 years: HR 4.12, 95% CI 2.18–7.78; ≥60 years: HR 2.04, 95% CI 1.48–2.81), as did non-obese stroke cases (BMI < 28: HR 1.74, 95% CI 1.31–2.31; Fig. 3).

### Sensitivity analysis

Upon conducting sensitivity analysis, we found that the increased risk of HF following new-onset stroke remained significant even after excluding participants with missing covariate data, those with a history of malignancy, and those using antihypertensive, hypoglycemic, or lipid-lowering medications (adjusted hazard ratio [aHR] 2.52, 95% confidence interval [CI] 1.89–3.38; aHR 2.73, 95% CI 2.04–3.64; aHR 2.39, 95% CI 1.58–3.62; aHR 2.30, 95% CI 1.69–3.11; aHR 2.12, 95% CI 1.55–2.90, respectively; see Supplemental Table 1). Additionally, we matched stroke patients with controls (1:4) based on propensity score, taking into account variables such as sex, age, current smoking, current drinking, high salt diet, physical activity, job nature, income, BMI, triglycerides, low-density lipoprotein cholesterol, C-reactive protein, use of antihypertensive drugs, lipid-lowering drugs, hypoglycemic drugs, history of myocardial infarction, history of atrial fibrillation, hypertension, and diabetes. Even after this matching, the risk of hip fracture remained significantly higher in stroke cases than in controls (adjusted hazard ratio 3.50, 95% CI 2.61–4.70; see Supplemental Table 2). Finally, the results of the mortality competition risk model showed the risk of HF remained higher in stroke cases than in controls (adjusted HR 2.44, 95% CI 2.32–2.56; Supplemental Table 3). The results of the study were consistent with the main analysis.

## Discussion

This is a large prospective study conducted to investigate the effect of new-onset stroke on hip fracture by tracking people from the time of diagnosis in China. Our study revealed that the risk of hip fracture was higher among stroke patients in the northern community of China, especially women, people under 60 years of age and non-obese people.

After adjustment for potential confounders, we found that the risk of HF was 2.35-fold higher in stroke cases than in controls (Table 2). Our findings are congruent with previous studies. A meta-analysis from China [24] suggested that stroke independently increased the risk of HF (adjusted risk ratio 2.06, 95% CI 1.68–2.52). A case-control study [25] also reported an increased risk of HF in the stroke population (adjusted odds ratio 1.96, 95% CI 1.65–2.33). Furthermore, a study in Taiwan found that the risk of HF was higher in participants with stroke than in the general population (adjusted HR 1.65, 95% CI 1.41–1.93) [9]. In addition, We also found an interaction effect between the risk of HF and age(*p* < 0.05). Further analyses with stratification for age showed that the risk of HF in stroke cases was increased by 4.12-fold in those aged < 60 years and by 2.04-fold in those aged ≥ 60 years in comparison with controls (Fig. 3). Based on age-stratified results, our findings are consistent with those of the case-control [25] and cohort studies [9] discussed above. However, our results could be considered more stable and reliable because we adjusted for confounders that were not adjusted for in the previous studies, including medications [9, 24] and physical activity, alcohol consumption, smoking, and BMI [9, 24, 25].

In our study, the risk of HF in stroke cases was increased by 3.10-fold in women and by 2.23-fold in men (Fig. 3). This finding is consistent with the results of a meta-analysis by Yuan et al. [6] and those of a prospective longitudinal study by Wu et al. [26], in which the risk of HF was higher in both female stroke cases (HR 2.33, 95% CI 1.62–3.34) and male stroke cases (HR 1.73, 95% CI 1.12–2.68) when compared with controls.

A recent meta-analysis that included studies from Japan, Europe, and North America [27] reported that stroke patients with a low BMI had an increased risk of HF. Specifically, compared with stroke patients who were overweight (BMI 25), those with a BMI of 20 had an increased risk of HF (risk ratio 1.95, 95% CI 1.71–2.22). However, being obese (BMI 30) was associated with a 17% decrease in risk of HF (risk ratio 0.83, 95% CI 0.69–0.99). In our study, the risk of HF was increased by 74% in non-obese stroke cases (BMI < 28) whereas obese (BMI ≥ 28) stroke cases and controls showed no significant increase in risk of HF (Fig. 3). Our finding with regard to the risk of HF in obese stroke cases is inconsistent with the results of the above-mentioned and may reflect differences in the study populations and the relatively small number of obese participants (n = 7,366) in this study.

In addition, the levels of individual indicators may abnormal under the history of malignant tumors [28]. And Treatment with antihypertensive [29], hypoglycemic [30], or lipid-lowering medication [31] might have had an effect on our study outcomes. However, the sensitivity analysis showed that removal of the above populations did not affect the final results, indicating that our findings are robust. Furthermore, we found that the higher risk of HF in stroke cases was independent of treatment with antihypertensive, hypoglycemic, or lipid-lowering medication and history of malignancy (Supplemental Table 1). In addition, the results from propensity score analysis was also consistent with the main analysis in this study. (Supplemental Table 2). After adjusting for the competing risk of all-cause mortality, the risk of HF was 2.44-fold higher in stroke cases than in controls (Supplemental Table 3). The results of this analysis are consistent with our main findings.

After excluding the potential influence of drugs and reducing the impact of confounding factors, such as comorbidities, our study still found that the risk of hip fracture after stroke remained significantly higher than that of the control group. This finding suggests that the increased incidence of hip fracture following stroke may be primarily attributed to falls rather than the effects of comorbidities or medication use. Additionally, our study found that falls were a common cause of hip fracture, which is consistent with previous research by Ramnemark et al. [32] who reported that falls were responsible for 84% of all fractures after stroke, with hip fracture being the most common type. These findings support our main results. However, the mechanism via which the risk of HF increases following stroke is still unclear, there are two possible explanations. The first is a post-stroke decrease in bone mineral density [33], which has been associated with a longer time confined to bed [34], use of warfarin anticoagulation [35], low BMI [27], severity of hemiplegia [36], and, in women, a decreased estrogen level [37]. On the side affected post-stroke, when hemiplegia is accompanied by loss of walking ability, loss of muscle mass owing to reduced mechanical load on the bones contributes to bone loss and an increased number of osteoclasts. It has been reported that motor impairment on the affected side results in an approximate 12–17% decline in bone density at one year post-stroke [38]. Moreover, decreased exposure to sunlight and subsequent vitamin D deficiency can lead to secondary hyperparathyroidism. Hyperthyroidism causes calcium and bone metabolism disorders, leading to type II osteoporosis [39]. Osteoporosis is highly prevalent in postmenopausal women owing to a combination of independent risk factors, including hormone deficiency, calcium loss, and aging [40].

The second explanation for the increased risk of HF following stroke is the increased risk of falls [39]. Motor function decreases on the hemiplegic side because of abnormal mechanical properties of skeletal muscle after stroke and is accompanied by decreased bone mineral density. Mobility is limited in the acute phase of stroke. However, non-elderly patients have greater mobility after stroke, and HF is more likely in these patients without measures to prevent falls. There is also a link between low BMI and muscle weakness attributable to nutrient deficiency, such as malnutrition or vitamin D deficiency [41], which leads to reduced stability of the greater trochanter and a greater risk of falling. Furthermore, falls are associated with post-stroke status, balance disorders, neglect, aphasia, use of diuretics, antidepressants, or sedatives, polypharmacy, history of falls, and other factors [42, 43].

To our knowledge, this is the first population-based cohort study to demonstrate the effect of stroke on the risk of HF in the mainland population of China. However, this study has some limitations. First, more information would be useful but may not be available. We had no information on bone mineral density or concomitant immune diseases such as hyperthyroidism or systemic lupus erythematosus. Therefore, it was not possible to evaluate the relationship between the risk of HF and the rate of bone loss after stroke, bone mineral density, and immune disea. Furthermore, the lack of information on osteoporosis treatment drugs prevented us from studying the role they may have played as a factor. Second, because of the relatively small number of hip fractures it may not be possible to due subset analyses. Moreover, it was not always confirmed whether hemiplegia was present post-stroke. Thus, we could not ascertain whether HF is more likely to occur on the hemiplegic side. Third, the study was based on the Kailuan study population and included a high proportion of men. Therefore, our results will need to be confirmed in other populations. However, despite these limitations, our findings are based on a large study population in China with detailed information available on stroke and associated confounders. Long-term follow-up was possible using management data and the exact causes of HF were known, which avoided any information bias stemming from unknown causes of HF. As a result, the research results still have certain guiding significance.

## Conclusions

In this study, stroke patients had a 2.35-fold higher risk of HF than the general population. The risk increased to 3.10-fold in women and 4.12-fold in those under 60 years of age and 1.74-fold in non-obese individuals. Therefore, health education is required to improve awareness of the risk of HF after stroke. Particularly in women, patients younger than 60 years, and those who are non-obese.

## Prevention strategy

Preventing hip fractures in post-stroke patients can be approached from two primary perspectives: preventing bone loss and reducing the risk of falls [44]. To prevent bone loss, non-pharmacological measures such as adequate sunlight exposure, early physiotherapy, and pharmacological measures such as oral and intravenous bisphosphonates, or calcium and vitamin D supplementation for hemiplegic patients may be considered [45]. Physical exercise can also be beneficial in improving muscle strength and cardiorespiratory fitness, which in turn, may improve bone health in chronic stroke patients [46]. Additionally, previous studies have reported a 30-fold increase in the risk of hip fracture in the event of a direct impact to the hip [47–48]. Therefore, the use of hip protectors that act as shock absorbers can be effective in preventing hip fractures caused by falls. These preventive measures may be necessary to minimize hip fractures in stroke patients during and after stroke rehabilitation.

## Electronic supplementary material

Below is the link to the electronic supplementary material.

Supplementary Material 1

## Data Availability

The datasets generated and/or analyzed during the current study are not publicly available but are available from the corresponding author on reasonable request.

## Abbreviations

HF: Hip fracture
BMI: Body mass index
HR: Hazard ratio
FBG: Fasting blood glucose
HDL-C: High-density lipoprotein cholesterol
LDL-C: Low-density lipoprotein cholesterol
hs-CRP: High-sensitivity C-reactive protein
TG: Triglyceride
TC: Total cholesterol
