# Supplementary material for: New-onset stroke on the risk of hip fracture: the Kailuan cohort study in China
Source: BMC Public Health. 2023 May 22;23:925. doi: 10.1186/s12889-023-15787-5 (PMC10204337; doi:10.1186/s12889-023-15787-5)
Supplement: Supplementary file 1 — Supplementary Material 1 [file 12889_2023_15787_MOESM1_ESM.doc]

**New-onset stroke on the risk of hip fracture: the Kailuan cohort study in China**

Nan Zhang^1^, Lu Guo^2^, Yaohui Yu^2^, Shuohua Chen^3^, Lishu Gao^4^, Xiaoli Hou^2^, Faming Tian^2*^, Shouling Wu^3*^

^1^ Department of orthopedics, Kailuan General Hospital, Tangshan, Hebei, China.

^2^ the School of Public Health, North China University of Science and Technology. Tangshan, Hebei, China.

^3^ Department of Cardiology, Kailuan General Hospital, Tangshan, Hebei, China.

^4^ Department of Endocrinology, Tangshan People’s Hospital, Tangshan, Hebei, China.

^*^Corresponding author: Shouling Wu, E-mail: drwusl@163.com;

Co-corresponding author: Faming Tian, E-mail: tfm9911316@163.com;

Co-first author: Lu Guo;

Contributing authors E-mail: guolu1013@163.com; yuyaohui1114@163.com;

csh01062011@163.com; gaolishu2022@126.com; houxiaoli1981@163.com;

**Supplemental Materials**

Supplemental table 1. Sensitivity Analysis:Hazard Ratios and 95% Confidence Intervals for the Incidence of HF for Stroke

patients Compared With the Matched controls.

Supplemental table 2. Sensitivity Analysis: Hazard Ratios and 95% Confidence Intervals for the Incidence of hip fracture for

Stroke patients Compared With the Matched controls(propensity score matching).

Supplemental table 3. Hazard Ratios and 95% Confidence Intervals for the Incidence of HF for Stroke patients Compared With the

Matched controls(Competing risk models for death)

**Supplemental table 1. Sensitivity Analysis:Hazard Ratios and 95% Confidence Intervals for the Incidence of HF for Stroke**

**patients Compared With the Matched controls**

|  | Events/Participants  （N） | Incidence rate  (per1000 PYs) | Model 1 | Model 2 | Model 3 |
| --- | --- | --- | --- | --- | --- |
|  |  |  | HR(95%CI) *p* value | HR(95%CI) *p* value | HR(95%CI) *p* value |
| Removal of missing covariates data |  |  |  |  |  |
| Controls  Stroke patients | 137/31192  73/7798 | 0.49  1.14 | 1.00  2.51(1.88-3.34)<0.001 | 1.00  2.51(1.87-3.36)<0.001 | 1.00  2.52(1.89-3.38)<0.001 |
| Removal of antihypertensive  drugs treatment |  |  |  |  |  |
| Controls | 67/15912 | 0.47 | 1.00 | 1.00 | 1.00 |
| Stroke patients | 35/3978 | 1.09 | 2.38(1.58-3.59)<0.001 | 2.38(1.57-3.60)<0.001 | 2.39(1.58-3.62)<0.001 |
| Removal of hypoglycemic  drugs treatment |  |  |  |  |  |
| Controls  Stroke patients | 133/30388  66/7597 | 0.48  1.06 | 1.00  2.29(1.70-3.09)<0.001 | 1.00  2.29(1.69-3.10)<0.001 | 1.00  2.30(1.69-3.11)<0.001 |
| Removal of lipid-lowering  drugs treatment |  |  |  |  |  |
| Controls  Stroke patients | 132/26836  60/6709 | 0.55  1.11 | 1.00  2.17(1.60-2.95)<0.001 | 1.00  2.12(1.55-2.90)<0.001 | 1.00  2.12(1.55-2.90)<0.001 |
| Removal of the history of  malignant tumors |  |  |  |  |  |
| Controls  Stroke patients | 136/33596  77/8399 | 0.45  1.12 | 1.00  2.72(2.05-3.60)<0.001 | 1.00  2.72(2.03-3.62)<0.001 | 1.00  2.73(2.04-3.64)<0.001 |

*Note: HR and 95%CI of HF were calculated in the stroke patients compared to controls; incidence density rate=number of incident cases/

person-years × 1000; PY, person-years; HR, hazard ratio. Model 1: adjusted for current smoking, current drinking, high salt diet, physical activity,

job nature, income, and BMI. Model 2: adjusted for model 1 plus triglycerides, low-density lipoprotein cholesterol*,* C*-*reactive protein, use of

antihypertensive drugs, use of lipid-lowering drugs, and hypoglycemic drugs. (no adjusted after excluding those populations). Model 3: adjusted

for model 2 plus history of myocardial infarction, and history of atrial.

**Supplemental table 2. Sensitivity Analysis: Hazard Ratios and 95% Confidence Intervals for the Incidence of hip fracture**

**for Stroke patients Compared With the Matched controls(propensity score matching)**

|  | Events/Participants  （N） | Follow-up duration  (PYs) | Incidence rate  (per1000 PYs) | Model |
| --- | --- | --- | --- | --- |
|  |  |  |  | HR(95%CI)*p* value |
| Controls | 105/32874 | 307542 | 0.51 | 1.00 |
| Stroke patients | 77/8285 | 68047 | 1.12 | 3.50(2.61-4.70)<0.001 |

*Note: HR and 95%CI of hip fractures were calculated in the stroke patients compared to controls; incidence density rate=number of incident

cases/person-years × 1000; PY, person-years; HR, hazard ratio. Model:crude model.

**Supplemental table 3. Hazard Ratios and 95% Confidence Intervals for the Incidence of HF for Stroke patients Compared With the**

**Matched controls(Competing risk models for death)**

|  | Model 1 | Model 2 | | | Model 3 |
| --- | --- | --- | --- | --- | --- |
|  | HR（95%CI）*p* value | HR（95%CI）*p* value | HR（95%CI）*p* value | | |
| Controls | 1.00 | 1.00 | | 1.00 | |
| Stroke patients | 2.33（2.22-2.45）<0.001 | 2.42（2.30-2.54）<0.001 | | 2.44（2.32-2.56）<0.001 | |

*Note: HR and 95%CI of hip fractures were calculated in the stroke patients compared to controls; HR, hazard ratio.Model 1: adjusted for current smoking,

current drinking, high salt diet, physical activity, job nature, income, and BMI.Model 2: adjusted for model 1 plus triglycerides, low-density lipoprotein

cholesterol*,* C*-*reactive protein, use of antihypertensive drugs, lipid-lowering drugs, and hypoglycemic drugs. Model 3: adjusted for model plus history of

myocardial infarction, and history of atrial fibrillation.
